# Structured sonic tube with carbon nanotube-like topological edge states

**DOI:** 10.1038/s41467-022-32777-0

**Published:** 2022-08-30

**Authors:** Zhiwang Zhang, Penglin Gao, Wenjie Liu, Zichong Yue, Ying Cheng, Xiaojun Liu, Johan Christensen

**Affiliations:** 1 Department of Physics, MOE Key Laboratory of Modern Acoustics, Collaborative Innovation Center of Advanced Microstructures, Nanjing University, 210093 Nanjing, China; 2 State Key Laboratory of Mechanical System and Vibration, School of Mechanical Engineering, Shanghai Jiao Tong University, 200240 Shanghai, China; 3 Institute of Vibration, Shock and Noise, Shanghai Jiao Tong University, 200240 Shanghai, China; 4 Department of Physics, Universidad Carlos III de Madrid, ES-28916 Leganés, Madrid Spain; 5 College of Aerospace Engineering, Chongqing University, 400044 Chongqing, China; 6 IMDEA Materials Institute, Calle Eric Kandel, 2, 28906 Getafe, Madrid Spain

**Keywords:** Acoustics, Topological insulators

## Abstract

A single-wall carbon nanotube can be viewed as a one-dimensional material created by rolling up a sheet of graphene. Its electronic band structure depends on the chirality, i.e., how the sheet has been rolled up, yet synthesizing the symmetry at will is rather challenging. We structure an artificial honeycomb lattice in both a zigzag and an armchair tube and explore their topological features for sound. Our findings reveal how armchair tubes remain gapless, whereas the zigzag counterparts host nontrivial edge states of non-zero quantized Zak phase, which are dictated by the circumferential number of units. Unlike man-made planar lattices whose underling symmetry must be broken to harvest quantum Hall and pseudospin phases, interestingly, the structured tubular lattice symmetry remains intact, while its nontrivial phase alone is governed by the chirality and the tube diameter. We foresee that our results, not only for sound, but also in photonics, mechanics and electronics will broaden future avenues for fundamental and applied sciences.

## Abstract (web summary)

Single-wall carbon nanotubes are made of carbon with diameters less than 100 nanometers. Here, the authors engineer an analogue tube with a diameter 1,000,000 times larger with the aim to explore topological properties including unusual acoustic edge states.

## Introduction

In order for a sheet of graphene to be seamlessly rolled up into a tube, one needs to overlap two hexagons of the underlying lattice in the process of doing so^1,2^. The chiral vector connecting the centers of the two hexagons determines the geometrical symmetry of the tubular layout, i.e., the tube topology. It implies thus that metallic or semiconducting phases of the single-wall carbon nanotube (SWCNT) depend chiefly on the chiral index^3–5^. In the meantime, precise control over the shape, size, and chirality of graphene nanoribbons has made easier the exploration of topological phases of matter to access otherwise inaccessible symmetry-protected electronic states^6–9^.

Ever since topological quantum engineering has reached the realm of classical wave physics, a plethora of exotic topological phases have surfaced in the scope of robust and reflection-less sound^10–12^, light^13–16^, and vibrations^17^. Beyond earlier achievements to conquer classical analogies of the Chern insulator or the spin and valley (pseudospin) degrees of freedom, the latest efforts have also focused on higher-order topological insulators^18–20^ and the combination of non-Hermiticity and topology^21–24^. Based on highly flexible means to engineer wave-based topological structures, this frontier is expected to continue flourishing and to expand from contemporary condensed matter physics, as in the yet inconceivable case of unraveling topological sound in an analog SWCNT.

In this work, we demonstrate that a topological nontrivial phase can be engineered in such a structured tube despite the fact that the lattice symmetry remains unaltered. This is in stark contrast to well-known topological phases in acoustics^12^ that are brought forward through broken time-reversal, mirror, or inversion symmetry, which requires additional design sophistication and fabrication challenges. To the best of our knowledge, a tubular topological approach that is inspired by SWCNT physics, both in acoustics and optics, has yet not seen the light of day.

## Results

### Planar acoustic graphene sheet

**Fig. 1:**
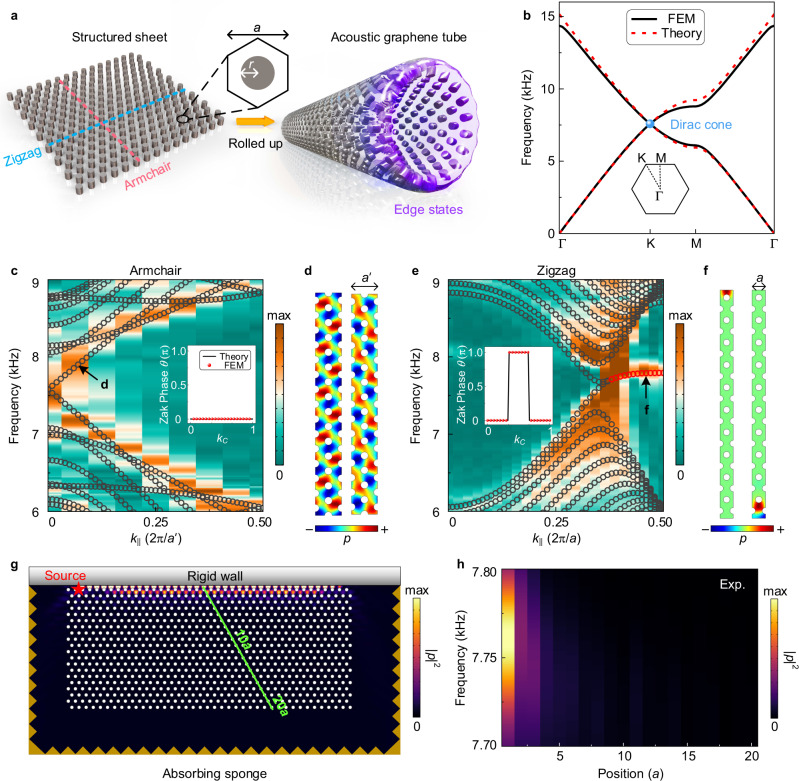
Topological properties of the acoustic graphene sheet. **a** Schematic of the AGT rolled up from a structured sheet. **b** Corresponding sheet band diagram calculated with the finite-element method (black solid curves) and theoretical predictions derived from the effective model (red dashed curves). Inset: the first Brillouin zone (BZ). **c** Simulated (black circles) and experimentally measured (background color) dispersion relation of the finite structured sheet along the AC direction. Inset: Zak phase. **d** Simulated eigen-profile of the degenerate bulk states highlighted in (**c**). **e**, **f** Same as **c**, **d** though along the ZZ direction supporting topological edge states [red circles in (**e**)] as shown in (**f**). **g** Schematic of the experimental setup with superimposed ZZ surface excitation. **h** Frequency-dependent sound intensity profile measured along the green line in (**g**).

We realize the acoustic counterpart of a SWCNT by rolling up an equivalent sheet made of a honeycomb arrangement of rigid rods (lattice constant and radius are *a* = 2.5 cm and *r* = 0.25*a*, respectively) as depicted in Fig. 1a. The physics of the acoustic graphene tube (AGT) can essentially be captured by its unrolled planar layout, which is why a topological analysis can be undertaken in the said geometry. In order to construct an effective acoustic Hamiltonian, we treat the sheet as a honeycomb waveguide network (see Supplementary Information for details). Thus, after solving the wave equation and applying Bloch’s theorem, we obtain the following acoustic eigenvalue problem

1 $${{{{{{{\mathcal{H}}}}}}}}({{{{{{{\bf{k}}}}}}}}){{{{{{{\boldsymbol{\psi }}}}}}}}=E{{{{{{{\boldsymbol{\psi }}}}}}}},$$

where $E=3\cos ({k}_{0}L)$, and ${{{{{{{\mathcal{H}}}}}}}}({{{{{{{\bf{k}}}}}}}})=[0,g({{{{{{{\bf{k}}}}}}}});{g}^{*}({{{{{{{\bf{k}}}}}}}}),0]$ with the off-diagonal term $g({{{{{{{\bf{k}}}}}}}})=\mathop{\sum }\nolimits_{l=1}^{3}\exp (-i{{{{{{{\bf{k}}}}}}}}\cdot {{{{{{{{\boldsymbol{\delta }}}}}}}}}_{l})$. The vectors ***δ***_*l*_ connect site A to its nearest neighboring site B (see Supplementary Information for details). Interestingly, Eq. (1) exactly maps into graphene considering the nearest-neighbor hopping effects only. By expanding *g*(**k**) in the vicinity of the *K* point, i.e., **k** = **K** + *δ***k**, the reduced Hamiltonian $\delta {{{{{{{{\mathcal{H}}}}}}}}}_{{{{{{{{\rm{D}}}}}}}}}$ reaches the standard massless Dirac formulation $\delta {{{{{{{{\mathcal{H}}}}}}}}}_{{{{{{{{\rm{D}}}}}}}}}(\delta {{{{{{{\bf{k}}}}}}}})={\upsilon }_{{{{{{{{\rm{D}}}}}}}}}(\delta {k}_{x}{{{{{{{{\boldsymbol{\sigma }}}}}}}}}_{x}+\delta {k}_{y}{{{{{{{{\boldsymbol{\sigma }}}}}}}}}_{y})$, where ***σ***_*x*_ and ***σ***_*y*_ are the Pauli matrices and ${\upsilon }_{{{{{{{{\rm{D}}}}}}}}}=\sqrt{3}\widetilde{a}/2$ is the Dirac velocity. The effective lattice period $\widetilde{a}$ represents the lattice constant of the effective waveguide network. With the relation $\widetilde{a} \; \approx \; 0.931a$, Fig. 1b displays a good agreement with numerical computations of the dispersion relation from the effective Hamiltonian.

**Fig. 2:**
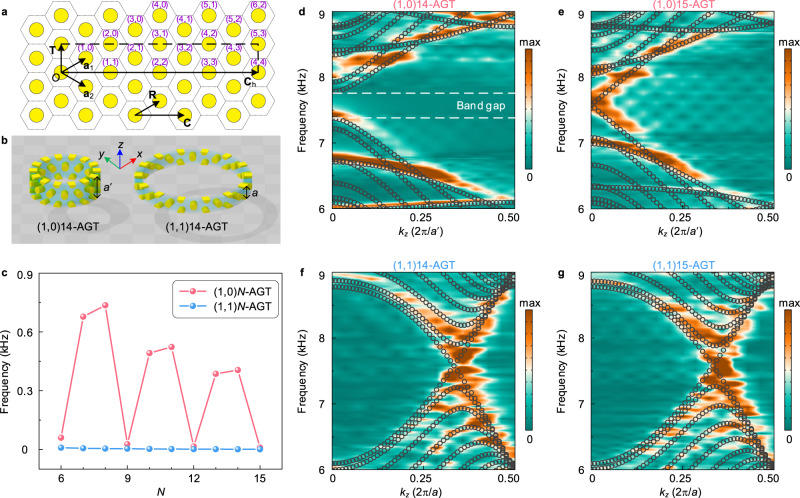
Sonic tubular dispersion engineering. **a** Chiral index map of the unrolled AGT, with topology-specific vectors for the armchair AGT. **b** Schematics of the unit cells of a (1, 0)14-AGT (left panel) and a (1, 1)14-AGT (right panel), which are periodic along the tube axis. **c** Gap width vs. circumferential units *N* for a (1, 0)*N*-AGT (red) and a (1, 1)*N*-AGT (blue). **d**, **e** Simulated (black circles) and measured (background color) dispersion relations for the (1, 0)*N*-AGT, with **d** *N* = 14 and **e** *N* = 15. *k*_*z*_ is the longitudinal wave vector along the tube axis. **f**, **g** Same as **d**, **e** but for the (1, 1)*N*-AGT.

The two achiral armchair (AC) and zigzag (ZZ) topologies host strikingly contrasting properties. In order to unravel this experimentally, we construct a structured sheet, i.e., a sonic crystal in a finite configuration as shown in Fig. 1g. Figure 1c displays the calculated (black circles) and measured (colored contour) AC gapless dispersion relation, whose Zak phase (inset) predicts a trivial topology. To match the sheet interface, we define a new set of lattice vectors as **C** and **R** with ∣**a**_1_ × **a**_2_∣ = ∣**C** × **R**∣ as shown in Fig. 2a. Accordingly, the Bloch wave vector is **k** = *k*_C_**c**_1_ + *k*_R_**c**_2_ with *k*_C,R_ ∈ [0, 1), where **c**_1_ and **c**_2_ are the reciprocal lattice vectors. To calculate the band structures of the structured sheet, we use a ribbon-shaped unit cell as shown in Fig. 1d and f, where the Floquet periodic conditions are imposed at the lateral boundaries. In this case, the structure can be considered as a one-dimensional sonic crystal with a period of *A*. Accordingly, the wave vector along the edge direction is defined as *k*_∣∣_. The relation between *k*_∣∣_ and *k*_C_ can be expressed as *k*_∣∣_ = 2*πk*_C_/*A* with *k*_C_ ∈ [0, 1), where $A=a^{\prime}=\sqrt{3}a$ for the AC edge and *A* = *a* for the ZZ edge. The corresponding degenerate eigenmode profiles (marked by a d in Fig. 1c) exhibit delocalized sound throughout the bulk. The measured dispersion relation was obtained through a Fourier transformation of the detected acoustic pressure fields (Methods). In contrast to the AC case, the ZZ interface hosts a non-zero quantized Zak phase that is accompanied by a topological non-trivial edge state as both experimental and numerical data show in Fig. 1e. Referring to this, Fig. 1f illustrates the computed surface excitations that are localized along the rigid boundary, which have also been confirmed with the finite-sheet computations where the ZZ interface adjacently boarders a rigid wall (Fig. 1g). The green line in this figure, marks the path along which the decaying intensity has been spectrally measured in Fig. 1h. Interestingly, the topologically protected edge state that has been launched by a point source at frequency *f* = 7.75 kHz, is not obtained by a prototypical breaking of the time-reversal, mirror, or inversion symmetry. Instead, the topology of the ZZ interface leads to a non-zero Zak phase whose continuous but finite width in momentum space, passes through the *K* or $K^{\prime}$ points of the one-dimensional (1D) BZ in an otherwise pristine lattice.

### Rolled-up structured sheet

The topology of the rolled-up AGT is determined by a chiral vector **C**_h_ = *N***C** = *n***a**_1_ + *m***a**_2_ and a translation vector **T** as shown in Fig. 2a. The greatest common divisor of the chiral index (*n*, *m*), denoted by *N* = gcd(*n*, *m*), represents the periodic numbers of the units along the circumference. Based on the six-fold symmetry of the underlying lattice, distinct tube geometries can be characterized by the integer pairs $(\hat{n},\hat{m})N$ with $0\le \hat{m}\le \hat{n}$. To illustrate the basic configurations under study, in Fig. 2b we depict the unit cells of two tubes, where the left panel shows the (1, 0)14-AGT with the ZZ edge and the right panel shows the (1, 1)14-AGT with the AC edge. Further, our numerical computations also show that the way the tubes are rolled up is not the only deciding ingredient to engineer a complete band gap. Its spectral width, as shown in Fig. 2c, appears to take discrete jumps with the number of circumferential unit cells. Specifically, for each unit number $N=3d,d\in {\mathbb{N}}$, the (1, 0)*N*-AGT remains gapless (red dots), whereas a band gap for the (1, 1)*N*-AGT never shows (blue dots) (see Supplementary Information for details). To give proof of this rule, we fabricate four AGTs with ZZ and AC edge terminations and measure their bulk dispersion. Two (1, 0)*N*-AGTs are constructed whose well-agreeing numerical and experimental band diagrams are shown in Fig. 2d and e. According to the rule in Fig. 2c, the measured (1, 0)14-AGT and (1, 0)15-AGT bands display clearly how a single circumferential increment of the units, leads to an acoustic semiconductor- and metal-like behavior, respectively. In other words, the (1, 0)14-AGT topology entails a complete band gap, whereas the (1, 0)15-AGT always remains gapless. In contrast, as shown in Fig. 2f and g, gapless dispersion relations have been observed for both AC tubes in accordance with the (1, 1)*N*-AGT predictions discussed in Fig. 2c.

### Observation of tubular edge states

**Fig. 3:**
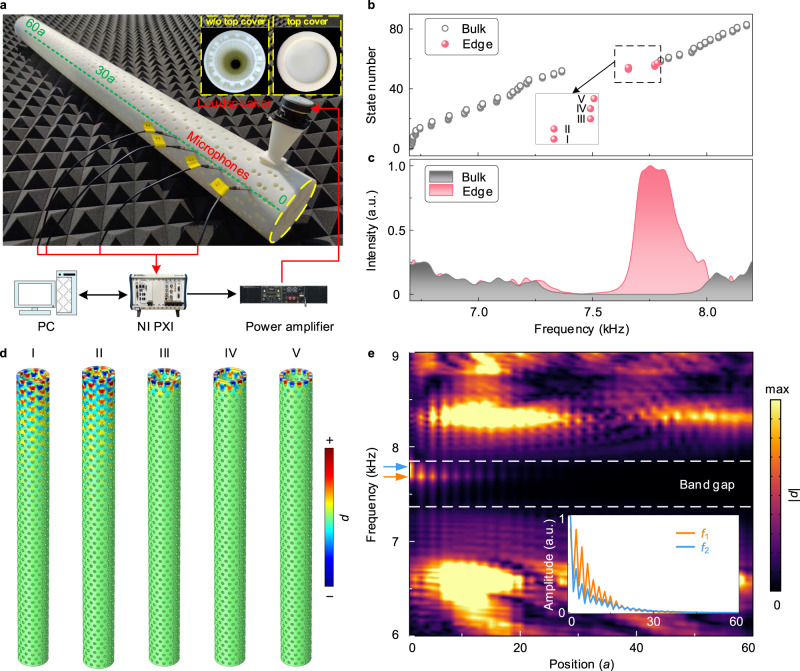
Topological edge states. **a** Photograph of the fabricated AGT. Insets: orifice without and with the top cover. **b** Calculated eigenfrequencies of the finite (1, 0)14-AGT. Gray circles and red dots represent the bulk and topological edge states, respectively. **c** Measured sound intensity spectra for those states. **d** Corresponding edge states profiles. **e** Frequency-dependent spatial profiles of the pressure fields measured along the tube axis [green dashed line in (**a**)]. Inset: magnification of the edge states at *f*_1_ = 7.688 kHz (orange) and *f*_2_ = 7.791 kHz (cyan).

In the tubular geometry, the topological picture changes in comparison to the sheet, in that only a discrete number of states fall into the nontrivial range of the band gap. As discussed earlier (Fig. 1), the nontrivial topological properties of the acoustic graphene sheet with a ZZ interface are guaranteed by the non-zero Zak phase in the range of 1/3 < *k*_C_ < 2/3, where the wavenumber *k*_C_ can take arbitrary values from 0 to 1. However, for the rolled-up AGT, the wavenumber is discretized to *N* values, induced by the inherent periodicity. Hence, the number of the topological edge states can be determined by

2 $${N}_{{{{{{{{\rm{edge}}}}}}}}}=\mathop{\sum }\limits_{\mu=0}^{N-1}|\upsilon ({k}_{{{{{{{{\rm{C}}}}}}}}})|,\,{{{{{{{\rm{with}}}}}}}}\,{k}_{{{{{{{{\rm{C}}}}}}}}}=\frac{\mu }{N}\,{{{{{{{\rm{and}}}}}}}}\,\mu \in [0,\; 1,\cdots \,,\; N-1],$$

where *υ*(*k*_C_) is the winding number (see Supplementary Information for details). A table with possible numbers of topological edge states in (1, 0)*N*-AGTs, can be found in Supplementary Information. Figure 3a shows a 3D-printed finite (1, 0)14-AGT containing 60 unit rings (Fig. 2b) that are stacked along the tube axis and terminated by a rigid cover. According to theory, five topological edge states can be expected to reside within the band gap (see Supplementary Information). At the nearest proximity to the tube termination, sound from a loudspeaker is funneled into the structured tube to excite the states under study, while condenser microphones are inserted at the designated positions to measure the local pressure fields (see the “Methods” section). At first, we calculate the eigenfrequencies of the AGT as illustrated in Fig. 3b. According to Eq. (2), five topological edge states (red dots) are found, which occupy the band gap spanning from 7.66 to 7.79 kHz, among a spectrum of bulk states (gray circles). Subsequently, we experimentally measure the spectral response of the detected acoustic intensity for those states, which in Fig. 3c are shown through colored peaks. In the midst of low-intensity waves that traverse the entire tubular geometry (gray), a noticeable (red) peak is observed in the spectrum that centers around 7.73 kHz, which stems from the adjoining said five edge states (I–V). Specifically, edge states I and II that are degenerate, whose eigenmodes as shown in Fig. 3d, exhibit acoustic edge confinement with considerable penetration lengths. The last three states (III–V), of which the former two are degenerate, on the other hand, display a much stronger localization at the termination where sound barely is capable to decay across two unit-rings. Lastly, to visualize the full scope of the topological tube, we scan the pressure field along the tube axis (green dashed line in Fig. 3a) in a frequency window from 6 to 9 kHz as shown in Fig. 3e. The bulk pressure hotspots resemble standing wave formations, but within the band gap, the adjoining five states around 7.73 kHz, display their edge localization by virtue of pressure attenuation along the tube. Indeed, as discussed in Fig. 3d, the two first degenerate states (I and II) at *f*_1_ = 7.688 kHz display weak edge confinement, in contrast to the highly concentrated edge states (III–V) at *f*_2_ = 7.791 kHz as the measured inset of Fig. 3e shows. Moreover, we further demonstrate that the AGT edge states can be remotely excited from a distant source despite being mixed with broadband white noise (see Supplementary Information).

## Discussion

We have demonstrated that SWCNT topology can be freely engineered in an analog acoustic setting, using rolled-up structured sheets of rigid rods. Our measurements reveal how acoustic tubes with ZZ terminations host nontrivial topological edge states in the audible range of around 7.7 kHz. We foresee that this geometry, but also other classical macroscale systems, presents an intriguing avenue to study unconventional topological quantum effects in a wave-based context, which may rise to the level of useful applications.

## Methods

### Numerical simulations

The numerical results presented in this work were implemented using the commercial finite-element-method simulation software COMSOL Multiphysics. The simulations were performed in the pressure acoustic module including the detailed structures with actual geometric dimensions. The background fluid is air with a mass density of *ρ* = 1.21 kg/m^3^ and a sound speed of *c* = 343 m/s. The boundaries of the 3D-printed cylinders have been modeled as hard-wall boundary conditions, owing to the large acoustic impedance mismatch between air and the printing materials of epoxy resin. The largest mesh element size was lower than one-tenth of the shortest incident wavelength. In the band-structure calculations, Floquet periodic boundary conditions were imposed at the boundaries of the periodic unit cells or strips. To calculate the Zak phase, the pressure field distributions of the eigenstates at specific wave vectors were extracted from the numerical eigenstate simulations. In the pressure-field calculations of the AGT, Fig. 3d for instance, the hard-wall boundary conditions were conducted at the top edge and the plane-wave radiation conditions were imposed at the bottom edge facing the air domain to eliminate interference from reflected waves. In this case, only the topological edge states confined around the top boundary exist.

### Experiments

The cylindrical rods of the acoustic graphene sheet and the rolled-up AGTs were precision-fabricated using epoxy resin via 3D printing. The fabricated sample in Fig. 1g consisted of 800 rods embedded in air and was covered by two plexiglass plates. The height of the rods was chosen to be 1.40 cm. In this scenario, the 2D approximation is applicable, since the planar waveguide supports propagating mode uniformly along the rod-axis for the wavelengths under consideration. Each AGT used in Figs. 2 and 3 were composed of 60 layers of units on the tube axis. In the experiments shown in Fig. 3, only one end of the AGT was terminated by a rigid cover to support the topological edge states, while the other end was exposed to free space, around which an absorbing sponge was placed to eliminate interference from reflected waves. Experiments were conducted by a loudspeaker (ENPILL PD-2121) with a 3D-printed pipe to generate a point sound source. Local pressure fields were measured by inserting a 1/4 in. condenser microphone (GRAS type 40PH) into the top plate at the designated positions. The outputs of the microphones were acquired by a digitizer (NI PXI-4498) and processed by LabVIEW program. Frequency scans were performed with an increment of 1 Hz. The dispersion relations of the bulk and boundary states in Figs. 1c, e and 2d–g were obtained by Fourier transforming the scanned acoustic pressure field distributions. The weak interference patterns in the measured dispersion relations are attributed to the finite-size effect of the structure, which can be reduced by increasing the scanning length of the Fourier transformation. The experimentally measured sound intensity of the edge states illustrated in Fig. 3c, was detected by the microphone placed near the rigid top cover and that of the bulk states was detected by the microphone placed in the AGT bulk which was far away from the boundary.

## Supplementary information

Supplementary Information

## Data Availability

The data that support the findings of this study are available from the corresponding authors on reasonable request.

## Code availability

All related codes can be built with the instructions in the "Methods” section. The codes for numerical simulations that support the findings of this study are also available from the corresponding authors on reasonable request.
