# Supplementary material for: Structured sonic tube with carbon nanotube-like topological edge states
Source: Nat Commun. 2022 Aug 30;13:5096. doi: 10.1038/s41467-022-32777-0 (PMC9428146; doi:10.1038/s41467-022-32777-0)
Supplement: Supplementary file 1 — Supplementary Information [file 41467_2022_32777_MOESM1_ESM.pdf]

Supplementary Information for:

Structured sonic tube with carbon nanotube-like topological edge states

Zhiwang Zhang,[1] Penglin Gao,[2,3,4] Wenjie Liu,[1] Zichong Yue,[1]

Ying Cheng,[1,*] Xiaojun Liu,[1,5,†] and Johan Christensen[4,6,‡]

[1]*Department of Physics, MOE Key Laboratory of Modern Acoustics,*

Collaborative Innovation Center of Advanced Microstructures,

Nanjing University, Nanjing 210093, China

[2]*State Key Laboratory of Mechanical System and Vibration,*

School of Mechanical Engineering, Shanghai Jiao Tong University, Shanghai, 200240, China

[3]*Institute of Vibration, Shock and Noise, Shanghai Jiao Tong University, Shanghai, 200240, China*

[4]*Department of Physics, Universidad Carlos III de Madrid, ES-28916 Leganés, Madrid, Spain*

[5]*College of Aerospace Engineering, Chongqing University, Chongqing 400044, China*

[6]*IMDEA Materials Institute, Calle Eric Kandel, 2, 28906, Getafe, Madrid, Spain*

[*] chengying@nju.edu.cn

[†] liuxiaojun@nju.edu.cn

[‡] jchriste@inst.uc3m.es

I. THEORETICAL MODELING OF ACOUSTIC GRAPHENE

We demonstrate that an acoustic graphene sheet can be effectively modeled as an acoustic honeycomb waveguide network and the effective Hamiltonian can be derived accordingly. As shown in Fig. S1a, the basic idea to build the effective model, consists in replacing the fluid regions among the rigid scatterers by junctions (sites A and B) that are connected to form the waveguide (narrow gray channels) network. In this way, giving the sound pressure p_{A} and particle velocity u_{A} at junction site A, the pressure p_{B} at its neighbouring site B can be expressed as [1, 2]

$$p_{\mathrm{B}} = \cos(k_0 L) p_{\mathrm{A}} + i \sin(k_0 L) \rho_0 c_0 u_{\mathrm{A}}, \tag{1}$$

where ρ_0 is the density of air, c_0 is the speed of sound, and $k_0 = \omega / c_0$ is the wavenumber with ω being the angular frequency. Considering the lattice site A in the unit cell, the above relation gives rise to the following set of equations

$$\begin{cases} p_{\mathrm{B}}^{(n-1,m)} = \cos(k_0 L) p_{\mathrm{A}}^{(n,m)} + i \sin(k_0 L) \rho_0 c_0 u_{\mathrm{A}}^1, \\ p_{\mathrm{B}}^{(n,m-1)} = \cos(k_0 L) p_{\mathrm{A}}^{(n,m)} + i \sin(k_0 L) \rho_0 c_0 u_{\mathrm{A}}^2, \\ p_{\mathrm{B}}^{(n,m)} = \cos(k_0 L) p_{\mathrm{A}}^{(n,m)} + i \sin(k_0 L) \rho_0 c_0 u_{\mathrm{A}}^3. \end{cases} \tag{2}$$

Here, the superscript index pair (n, m) marks the lattice site. Due to the conservation of the acoustic flow rate $\sum_{l=1}^{3} w u_{\mathrm{A}}^l = 0$, at the junction we obtain

$$3 \cos(k_0 L) p_{\mathrm{A}}^{(n,m)} = p_{\mathrm{B}}^{(n-1,m)} + p_{\mathrm{B}}^{(n,m-1)} + p_{\mathrm{B}}^{(n,m)}. \tag{3}$$

Equivalently, at lattice site B we obtain

$$3 \cos(k_0 L) p_{\mathrm{B}}^{(n,m)} = p_{\mathrm{A}}^{(n+1,m)} + p_{\mathrm{A}}^{(n,m+1)} + p_{\mathrm{A}}^{(n,m)}. \tag{4}$$

Applying Bloch's theorem thus gives rise to the eigenvalue problem

$$\mathcal{H}(\mathbf{k}) \boldsymbol{\psi} = E \boldsymbol{\psi}, \tag{5}$$

where $E = 3\cos(k_0 L)$, and $\mathcal{H}(\mathbf{k}) = [0, g(\mathbf{k}); g^*(\mathbf{k}), 0]$ with the off-diagonal term $g(\mathbf{k}) = \sum_{l=1}^{3} \exp(-i\mathbf{k} \cdot \boldsymbol{\delta}_l)$. Vector $\boldsymbol{\delta}_l$ connects site B to its nearest neighboring site A as shown in Fig. S1a. Eq. (5) defines an effective Hamiltonian of chiral symmetry that exactly maps to a graphene system considering the nearest-neighbor hopping effect only [3].

Next, we focus on the wave dynamics around the Dirac point. By expanding $g(\mathbf{k})$ in the vicinity of the K point, i.e., $\mathbf{k} = \mathbf{K} + \delta\mathbf{k}$, the reduced Hamiltonian $\delta\mathcal{H}_{\mathrm{D}}$ equates to the standard massless Dirac formulation

$$\delta\mathcal{H}_{\mathrm{D}}(\delta\mathbf{k}) = \upsilon_{\mathrm{D}}(\delta k_x\boldsymbol{\sigma}_x + \delta k_y\boldsymbol{\sigma}_y), \tag{6}$$

where $\boldsymbol{\sigma}_x$ and $\boldsymbol{\sigma}_y$ are the Pauli matrices, $\upsilon_{\mathrm{D}} = \sqrt{3}\widetilde{a}/2$ is the Dirac velocity with the effective lattice period $\widetilde{a} = |\mathbf{a}'_1| = |\mathbf{a}'_2|$. The massless Dirac Hamiltonian suggests linear crossing of the Dirac cones of the form $E = 3\cos(k_0 L) = \pm\upsilon_{\mathrm{D}}|\delta\mathbf{k}|$, which can be rewritten as

$$\begin{aligned} f &= f_{\mathrm{D}} + \delta f \\ &= \frac{c_0}{2\pi L}\arccos(\pm\frac{\upsilon_{\mathrm{D}}|\delta\mathbf{k}|}{3}) \\ &= \frac{c_0}{2\pi L}\left[\frac{\pi}{2} \mp \frac{\upsilon_{\mathrm{D}}|\delta\mathbf{k}|}{3} + O\left((\frac{\upsilon_{\mathrm{D}}|\delta\mathbf{k}|}{3})^3\right)\right], \end{aligned} \tag{7}$$

where δf measures the frequency increment of to the Dirac frequency f_{D}. Subsequently, the effective parameters of the acoustic network are given by

$$L = \frac{c_0}{4f_{\mathrm{D}}}, \quad \widetilde{a} = \frac{\sqrt{3}\pi}{f_{\mathrm{D}}}\frac{|\delta f|}{|\delta\mathbf{k}|}. \tag{8}$$

The band diagram of the artificial honeycomb lattice is calculated with the finite-element method (FEM) as depicted in main text Fig. 1b (black curves). The Dirac cone appears exactly at the Brillouin zone (BZ) corner. The values $f_{\mathrm{D}} \approx 7578.5$ Hz and $\delta f \approx 108.6$ Hz when $|\delta\mathbf{k}| \approx 3.35\,\mathrm{m}^{-1}$ have been derived from the numerical data. Consequently, we obtain $L \approx 0.453a$ and $\widetilde{a} \approx 0.931a$. With these effective parameters, the Hamiltonian formulation gives a nearly identical band diagram (red dashed curves) in comparison to the FEM results in Fig. 1b. The good agreement validates the effectiveness of the Hamiltonian and its preservation of the chiral symmetry of the acoustic lattice.

II. ONE-DIMENSIONAL LATTICE MODEL AND TOPOLOGICAL PROPERTIES OF AGTS

At first, a gapless graphene sheet seems unrelated to topological insulators and their gapped phases. By rolling up such sheet to a tube, however, it can actually be topologically nontrivial due to the discretization of wavenumbers. In this section, we carefully study how the designed AGT acquires topological nontrivial properties by mapping it to a 1D lattice with chiral symmetry.

Figure S1a shows a sketch of an effective acoustic graphene sheet cut into a large rectangle, which is then rolled up and fixed at the lateral ends. The circumference of the tube can be expressed by a chiral vector $\mathbf{C}_\mathrm{h} = n\mathbf{a}'_1 + m\mathbf{a}'_2$, where $\mathbf{a}'_1 = (\sqrt{3}/2, 1/2)\widetilde{a}$ and $\mathbf{a}'_2 = (\sqrt{3}/2, -1/2)\widetilde{a}$ are the primitive lattice vectors of the graphene sheet. The integer numbers, i.e., the chiral index (n, m) have the greatest common divisor N, which indicates the Nth-fold rotation symmetry around the tube axis. Beyond this, the tube also has helical symmetry along its axis. These two symmetries essentially stem from the translational symmetry of the graphene sheet described by a new set of lattice vectors, $\mathbf{C} = \mathbf{C}_\mathrm{h}/N = \hat{n}\mathbf{a}'_1 + \hat{m}\mathbf{a}'_2$ and $\mathbf{R} = p\mathbf{a}'_1 + q\mathbf{a}'_2$. As illustrated in Fig. S1a, $\mathbf{C}$ and $\mathbf{R}$ define a new unit cell in the 2D sheet, from which we obtain $\hat{m}p - \hat{n}q = 1$ from the identity $|\mathbf{a}'_1 \times \mathbf{a}'_2| = |\mathbf{C} \times \mathbf{R}|$. Based on the symmetry analysis, we conclude that the topological properties of the rolled-up tube are inherent to the pristine 2D graphene sheet given that longitudinal sound waves are almost insensitive to the curvature effect.

Next, we study the effective Hamiltonian of the acoustic graphene sheet $\mathcal{H}(\mathbf{k})$. By considering only the relative phase to neighbouring sites, the off-diagonal term can be expressed as [4]

$$g(\mathbf{k}) = 1 + e^{i\mathbf{k}\cdot(\boldsymbol{\delta}_3 - \boldsymbol{\delta}_2)} + e^{i\mathbf{k}\cdot(\boldsymbol{\delta}_3 - \boldsymbol{\delta}_1)}. \tag{9}$$

The Bloch wave vector $\mathbf{k}$ is the key ingredient that shall be expressed in accordance to the new lattice vectors $\mathbf{C}$ and $\mathbf{R}$ to match the boundaries of the AGT

$$\mathbf{k} = k_\mathrm{C}\mathbf{c}_1 + k_\mathrm{R}\mathbf{c}_2, \quad k_\mathrm{C,R} \in [0, 1). \tag{10}$$

As illustrated in Fig. S1b, here $\mathbf{c}_1$ and $\mathbf{c}_2$ are the reciprocal lattice vectors, which, based on $\mathbf{C}$ and $\mathbf{R}$, are defined as

$$\begin{cases} \mathbf{c}_1 = -q\mathbf{b}_1 + p\mathbf{b}_2, \\ \mathbf{c}_2 = \hat{m}\mathbf{b}_1 - \hat{n}\mathbf{b}_2, \end{cases} \tag{11}$$

where $\mathbf{b}_1 = 2\pi/\widetilde{a}(1/\sqrt{3}, 1)$ and $\mathbf{b}_2 = 2\pi/\widetilde{a}(1/\sqrt{3}, -1)$ are the reciprocal counterparts to $\mathbf{a}'_1$ and $\mathbf{a}'_2$. Substituting Eqs. (10) and (11) into Eq. (9) finally gives rise to

$$g(k_\mathrm{C}, k_\mathrm{R}) = 1 + e^{2\pi i(-pk_\mathrm{C} + \hat{n}k_\mathrm{R})} + e^{2\pi i(qk_\mathrm{C} - \hat{m}k_\mathrm{R})}. \tag{12}$$

We emphasize that k_C is associated with the Fourier transformation along the $\mathbf{C}$ direction. For a 2D acoustic graphene sheet it can take arbitrary values ranging from 0 to 1. The two-by-two effective Hamiltonian, $\mathcal{H}(\mathbf{k}) = [0, g(\mathbf{k}); g^*(\mathbf{k}), 0]$, describes a 1D lattice model when k_R runs through the 1D BZ for each k_C. Thus, the winding number can be characterized by

$$v(k_\mathrm{C}) = \frac{1}{2\pi i}\int_0^1 \frac{\partial \ln[g(k_\mathrm{C}, k_\mathrm{R})]}{\partial k_\mathrm{R}} dk_\mathrm{R}, \tag{13}$$

where $g(k_{\text{C}}, k_{\text{R}})$ traces out a closed curve on the complex plane, and $v(k_{\text{C}})$ counts the number of times it winds around the origin. In addition, the chiral symmetry also ensures a quantized Zak phase [5]

$$\theta(k_{\text{C}}) = \int_0^1 i \left\langle \boldsymbol{\psi}(\mathbf{k}) | \nabla_{k_{\text{R}}} \boldsymbol{\psi}(\mathbf{k}) \right\rangle dk_{\text{R}}, \tag{14}$$

which relates to the winding number through $\theta(k_{\text{C}}) = |v(k_{\text{C}})|\pi$. The theoretically calculated (black line) and the numerically derived (red dots) Zak phases of the acoustic sheets of different topologies, have been discussed in the insets of Figs. 1c and 1e (of the main text), which indicate a nontrivial topological phase for the zigzag-edge sheet, but a trivial one for the armchair-edge one. In the zigzag-edge case, $\theta(k_{\text{C}}) = \pi$ accounts for a topologically nontrivial phase for $1/3 < k_{\text{C}} < 2/3$. The borders correspond to the transition points when the 1D BZ passes through the reciprocal lattice sites K and K'.

However, for a rolled-up AGT, only several discrete values are allowed due to the introduction of the periodic boundary along the circumferential direction, which is given by

$$\mathbf{k} \cdot \mathbf{C}_{\text{h}} = 2\pi\mu, \quad \mu \in \mathbb{Z}. \tag{15}$$

Explicitly, k_{C} hosts only N values of equal interval:

$$k_{\text{C}} = \frac{\mu}{N}, \quad \mu \in [0, 1, \cdots, N-1]. \tag{16}$$

Next, we discuss the conditions required to gap/close the Dirac cone of the AGT. For the gapped phase, the K and K' points should be excluded from the discrete BZ as described by Eq. (16). As shown in Fig. S1b, the positions of the K and K' points can be denoted as

$$\mathbf{k}(K, K') = \left[\frac{2\pi}{\sqrt{3}a}(\alpha + \beta + 1), \frac{2\pi}{a}(\alpha - \beta \pm \frac{1}{3})\right], \quad \alpha, \beta \in \mathbb{Z}, \tag{17}$$

which when coinciding with the discrete BZ yield

$$\mu = N\left[\hat{n}\alpha + \hat{m}\beta + \frac{\hat{n} + 2\hat{m}}{3}\right], \quad \text{or} \quad \mu = N\left[\hat{n}\alpha + \hat{m}\beta + \frac{2\hat{n} + \hat{m}}{3}\right]. \tag{18}$$

Note that $(\hat{n}\alpha + \hat{m}\beta)$ always gives an integer number. To make μ an integer, it requires $N(\hat{n} + 2\hat{m})/3$ or $N(2\hat{n} + \hat{m})/3$ to be integer numbers too. In addition to these two cases, all other AGTs sustain complete band gaps. In particular, the condition of $\hat{m} = \hat{n} = 1$, which makes μ an integer, ensures the gapless phase for a rolled up tube with an armchair edge. But for a zigzag-edge AGT, μ becomes an integer only when $N = 3d$ ($d \in \mathbb{N}$) and it poses a restriction to the diameter of the

AGT. The above conclusions about the achiral armchair and zigzag cases have been thoroughly discussed in the main text Fig. 2c.

For a gapped AGT with a nontrivial topological phase, we demonstrate that the numbers of topological edge states (denoted as N_{edge}) depend on the numbers of the discrete k_{C} that fall into the topological nontrivial range. It can be expressed as

$$N_{\text{edge}} = \sum_{\mu=0}^{N-1} |\upsilon(k_{\text{C}})|, \quad \text{with} \quad k_{\text{C}} = \frac{\mu}{N}, \tag{19}$$

where $\upsilon(k_{\text{C}})$ is the winding number and is related to the Zak phase by $\theta(k_{\text{C}}) = |\upsilon(k_{\text{C}})|\pi$. Considering the calculated Zak phase of the zigzag-edge AGT from Fig. 1e and the discrete wavenumber in Eq. (16), we obtain the numbers of the topological edge states in finite (1,0)N-AGT as listed in Table I. Indeed the numbers of edge states can be simplified by taking the nearest integer of $N/3$ with $N \neq 3d, d \in \mathbb{N}$ for the ZZ AGT. E.g., for the of (1,0)14-AGT, we obtain five edge states in the band gap. Figure S2 illustrates the case of a (1,0)13-AGT where four edge states are predicted in the complete band gap, in agreement with the data shown in Table I. Figure S2a illustrates the unit cell of the (1,0)13-AGT. The theoretically derived Zak phase of the zigzag-edge acoustic graphene sheet is shown by the solid curve in Fig. S2b together with the discrete BZ labelled by the equidistant lines, which is induced from the periodic conditions of the tube structure. According to Eq. (19), the number of the topological edge states, equals the number of the wavenumbers located in the topological nontrivial region [red dashed lines in Fig. S2b]. We further calculate the dispersion relation as plotted in Fig. S2c and a complete band gap appears due to the condition $N \neq 3d$, $d \in \mathbb{N}$. Consequently, four edge states can be found in this complete band gap as shown in the eigenfrequencies calculations (Fig. S2d). Figure S2e indicates the pressure field distributions of the four edge eigenstates, where the sound waves are tightly confined around the rigid boundary and decay into bulk rapidly. Without loss of generality, we also construct the chiral (2,1)N-AGT with $\hat{n} = 2$, $\hat{m} = 1$, $p = 1$ and $q = 0$ to further verify the above theory. We additionally study the case with $N = 10$ whose results are presented in Fig. S3. Here, three edge states are obtained according to both theory and simulations.

TABLE I: The number of topological edge states.

N	5	6	7	8	9	10	11	12	13	14	15
N_{edge}	2	×	2	3	×	3	4	×	4	5	×

III. BAND GAP ENGINEERING IN THE ZZ AND THE AC AGTS

With the aid of Fig. S4, we explain the band gap formation or the lack thereof with respect to the tube chirality. Figure S4a illustrates the schematic of the ZZ-edge acoustic graphene sheet, which is rolled up along the tube axis to form a (1,0)N-AGT. The corresponding reciprocal lattice is shown in Fig. S4b, together with the allowed wave vectors (red lines) for a (1,0)7-AGT. Since none of the discrete wave vectors pass through the K and K' points, a complete band gap is formed. Similarly, whenever $N \neq 3d, d \in \mathbb{N}$ for the ZZ AGT, the same gapped scenario prevails. However, AC (1,1)N-AGTs (Figs. S4c and S4d) remain gapless since the discrete wave vectors contain the K and K' points at all times independently of the choice of N, as the magnification in Fig. S4d clearly displays.

IV. ENHANCED SOUND SENSING IN A NOISY ENVIRONMENT

In this section, we provide an idea to use the AGT to pick up distant radiated acoustic signals that are masked by background noise. The concept is to let the AGT edge state be excited from a targeted source (with frequency $f_{\mathrm{T}} = 7.755$ kHz), while being heavily interfered by noise signals. Figure S5a illustrates the experimental setup comprising a (1,0)14-AGT of which the top lid contains perforation to ensure sound waves penetration. In Figs. S5b and S5c, measurements in free space display both in time and frequency that the targeted signal is entirely masked by the background noise. In contrast, when detecting the signal at the upper AGT edge, only a sharp signal at its edge state frequency prevails, while the remaining amplitude spectrum is free from unwanted noise (Figs. S5d and S5e). We consider this to be an interesting avenue combining both contemporary condensed matter physics and applied acoustics.

[1] C. Depollier, J. Kergomard, and J. Lesueur, J. Sound Vib. **142**, 153 (1990).

[2] L.-Y. Zheng, V. Achilleos, O. Richoux, G. Theocharis, and V. Pagneux, Phys. Rev. Applied **12**, 034014 (2019).

[3] A. C. Neto, F. Guinea, N. M. Peres, K. S. Novoselov, and A. K. Geim, Rev. Mod. Phys. **81**, 109 (2009).

[4] R. Okuyama, W. Izumida, and M. Eto, Phys. Rev. B **99**, 115409 (2019).

[5] J. K. Asbóth, L. Oroszlány, and A. Pályi, Lecture Notes in Physics **919**, 166 (2016).

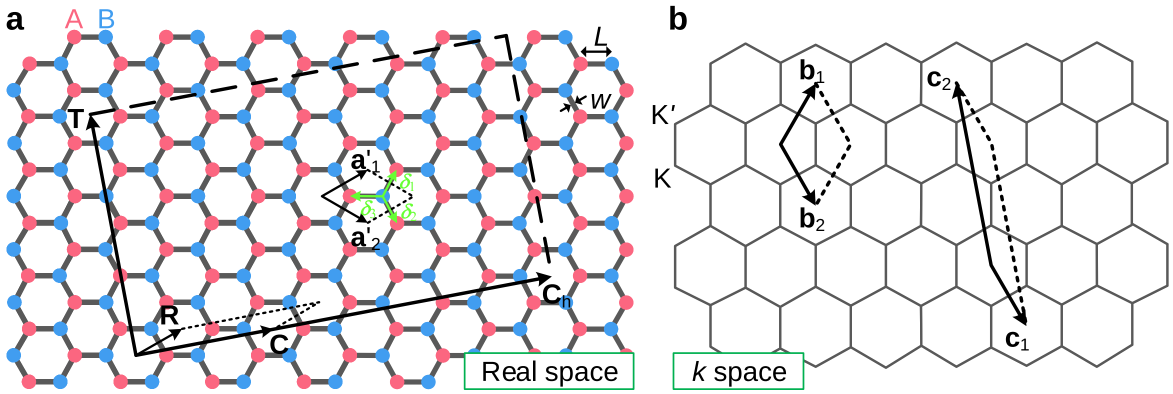

FIG. S1: **a**, Real space and **b**, reciprocal space of the effective model of the acoustic graphene sheet.

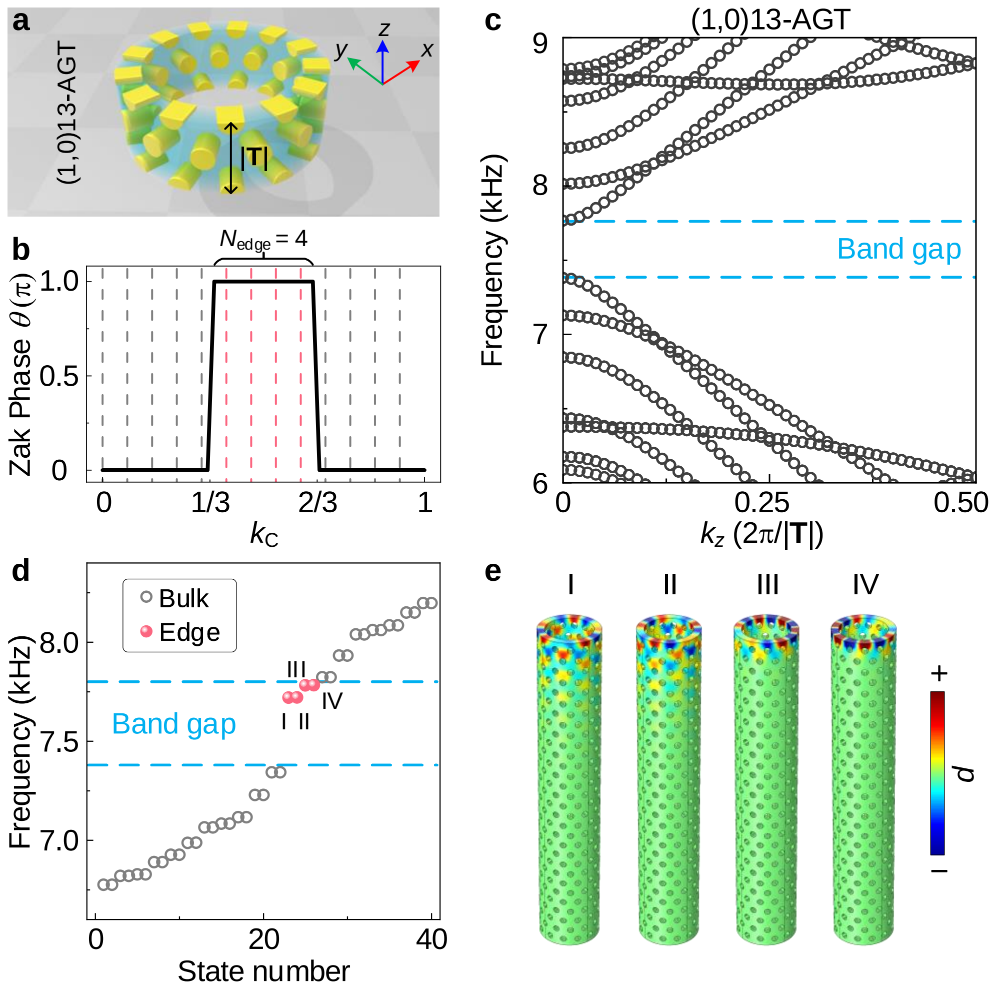

FIG. S2: **Topological properties of the finite (1, 0)13-AGT. a**, Schematic of the unit cell. The period along the tube axis is labelled by $|\boldsymbol{T}|$. **b**, Calculated Zak phase (black solid curve) and the discrete BZ (dashed lines). The wavenumber located in the topological nontrivial region is marked by the red dashed line. **c**, Simulated dispersion relation for the (1, 0)13-AGT. The region between the cyan lines represents the complete band gap. **d**, Calculated eigenfrequencies of the finite (1, 0)13-AGT. Gray circles and red dots represent the bulk and topological edge states, respectively. **e**, Corresponding acoustic modes of the four edge states labelled in (**d**).

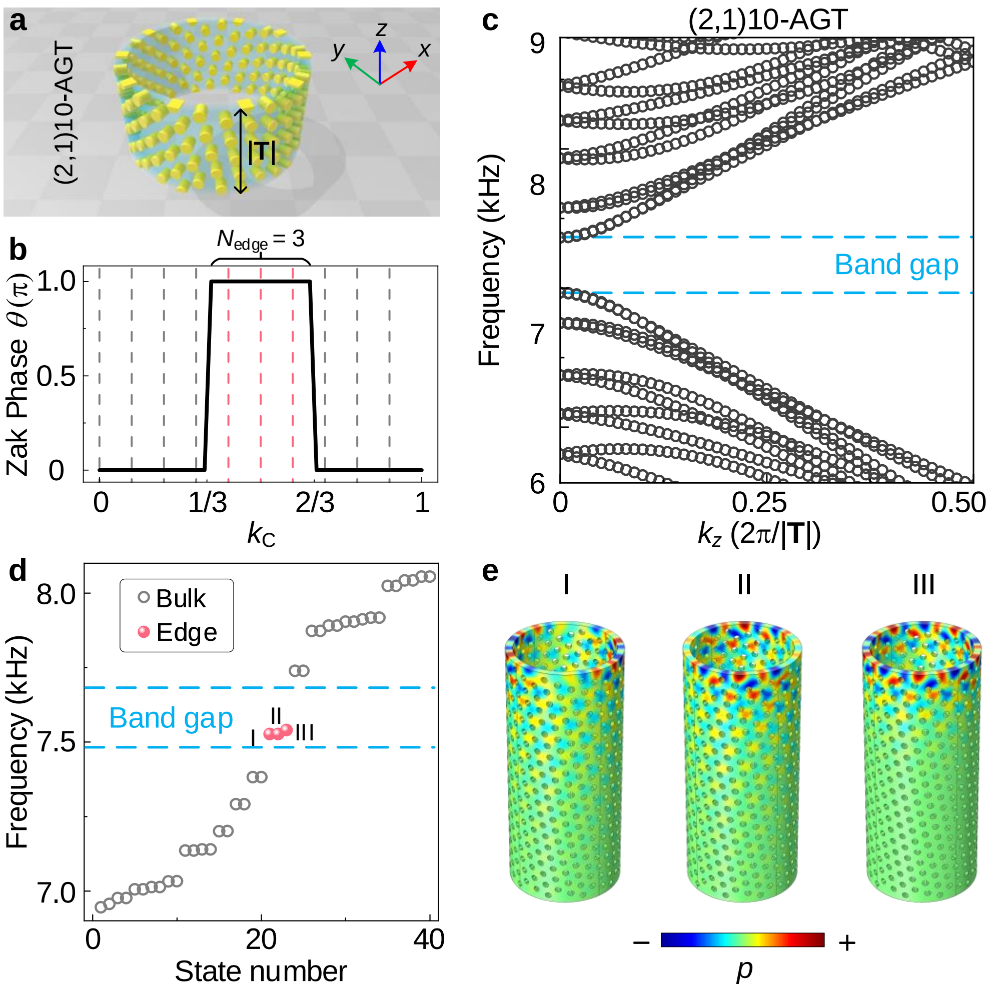

FIG. S3: **Topological properties of the finite (2, 1)10-AGT.** **a**, Schematic of the unit cell. **b**, Calculated Zak phase (black solid curve) and the discrete BZ (dashed lines). **c**, Simulated dispersion relations for the (2, 1)10-AGT. **d**, Calculated eigenfrequencies of the finite (2, 1)10-AGT. **e**, Corresponding acoustic modes of the three edge states labelled in (**d**).

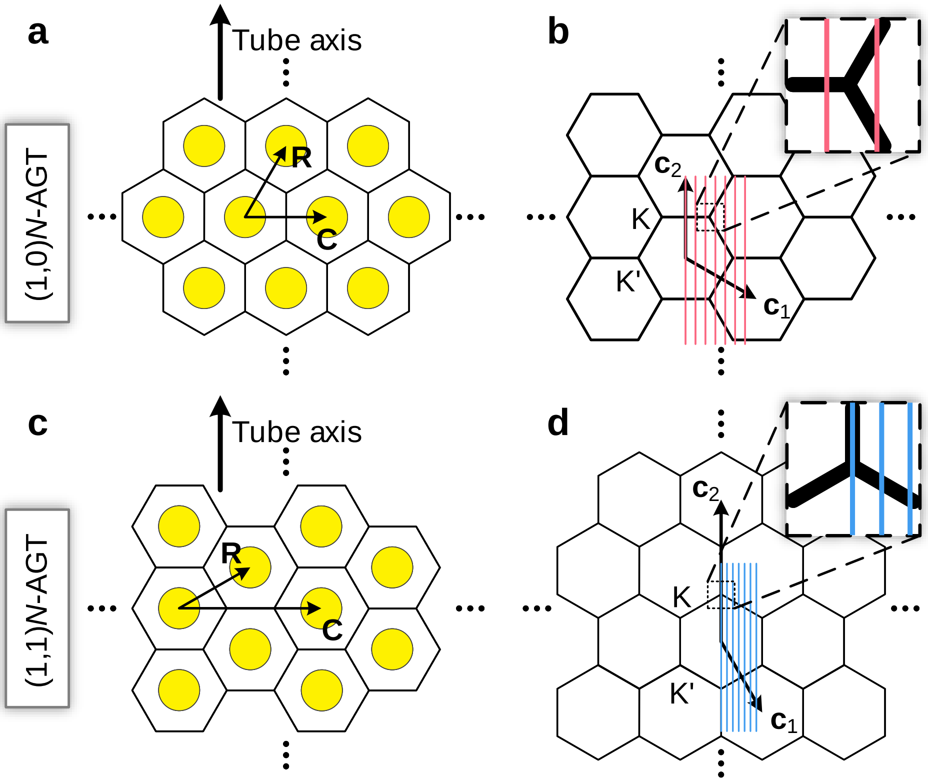

FIG. S4: **Band gap engineering in AGTs. a**, Geometric configuration for the (1,0)N-AGT. **b**, The reciprocal lattice of an acoustic graphene sheet (black hexagons) and the wave vectors allowed by the inherent periodic boundary condition along the circumferential direction (red lines) for the (1,0)7-AGT. **c**,**d**, Same as (**a**),(**b**) but for the (1,1)N-AGT. Blue lines in (**d**) represent the allowed wave vectors for the (1,1)7-AGT.

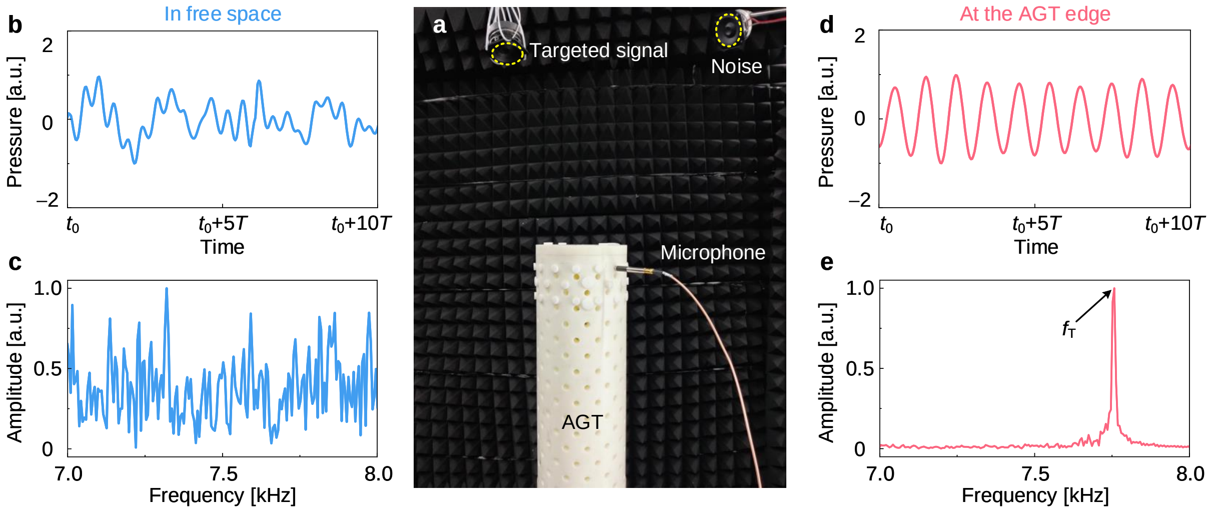

FIG. S5: **Enhanced sound sensing in a noisy environment.** **a**, Experimental setup: the targeted sinusoidal signal is emitted from a loudspeaker with the frequency $f_{\rm T} = 7.755$ kHz. The interference source radiates broadband white noise to mask the targeted signal. A microphones is placed at the upper edge of the AGT. **b**, Pressure signal in the time domain and **c**, the corresponding measured fast-Fourier-transform of the signal in free space without the AGT. **d**,**e**, Same as (**b**),(**c**) but measured at the AGT edge.
